# Celecoxib alleviates tamoxifen-instigated angiogenic effects by ROS-dependent VEGF/VEGFR2 autocrine signaling

**DOI:** 10.1186/1471-2407-13-273

**Published:** 2013-06-03

**Authors:** B N Prashanth Kumar, Shashi Rajput, Kaushik Kumar Dey, Aditya Parekh, Subhasis Das, Abhijit Mazumdar, Mahitosh Mandal

**Affiliations:** 1 School of Medical Science and Technology; Indian Institute of Technology Kharagpur, Kharagpur-721302, West Bengal, PIN-721302, India; 2 Department of Clinical Cancer Prevention, University of Texas MD Anderson Cancer Centre, Houston, TX, USA

## Abstract

**Background:**

Tamoxifen (TAM) is widely used in the chemotherapy of breast cancer and as a preventive agent against recurrence after surgery. However, extended TAM administration for breast cancer induces increased VEGF levels in patients, promoting new blood vessel formation and thereby limiting its efficacy. Celecoxib (CXB), a selective COX-2 inhibitor, suppresses VEGF gene expression by targeting the VEGF promoter responsible for its inhibitory effect. For this study, we had selected CXB as non-steroidal anti-inflammatory drug in combination with TAM for suppressing VEGF expression and simultaneously reducing doses of both the drugs.

**Methods:**

The effects of CXB combined with TAM were examined in two human breast cancer cell lines in culture, MCF7 and MDA-MB-231. Assays of proliferation, apoptosis, angiogenesis, metastasis, cell cycle distribution, and receptor signaling were performed.

**Results:**

Here, we elucidated how the combination of TAM and CXB at nontoxic doses exerts anti-angiogenic effects by specifically targeting VEGF/VEGFR2 autocrine signaling through ROS generation. At the molecular level, TAM-CXB suppresses VHL-mediated HIF-1α activation, responsible for expression of COX-2, MMP-2 and VEGF. Besides low VEGF levels, TAM-CXB also suppresses VEGFR2 expression, confirmed through quantifying secreted VEGF levels, luciferase and RT-PCR studies. Interestingly, we observed that TAM-CXB was effective in blocking VEGFR2 promoter induced expression and further 2 fold decrease in VEGF levels was observed in combination than TAM alone in both cell lines. Secondly, TAM-CXB regulated VEGFR2 inhibits Src expression, responsible for tumor progression and metastasis. FACS and in vivo enzymatic studies showed significant increase in the reactive oxygen species upon TAM-CXB treatment.

**Conclusions:**

Taken together, our experimental results indicate that this additive combination shows promising outcome in anti-metastatic and apoptotic studies. In a line, our preclinical studies evidenced that this additive combination of TAM and CXB is a potential drug candidate for treatment of breast tumors expressing high levels of VEGF and VEGFR2. This ingenious combination might be a better tailored clinical regimen than TAM alone for breast cancer treatment.

## Background

Extensive clinical studies over the past 30 years have shown that tamoxifen (TAM) can reduce the incidence and regression of breast carcinoma among women worldwide. A selective estrogen receptor (ER) modulator, TAM has been used extensively in the clinical management of primary and advanced breast cancer and is also widely employed as a preventive agent after surgery for breast cancer [1]. High survival rates for patients with early breast cancer as well as improved quality of life for patients with metastatic disease are observed in patients administered TAM. It also reduces the incidence of breast cancer in patients at risk for developing the disease and also the recurrence in women with ductal carcinoma in situ [2]. The constitutive therapeutic efficacy of TAM is due to its anti-proliferative action of binding competitively to ER, thereby blocking the mitogenic effect of estradiol [3].

Angiogenesis, a major attribute of tumorigenesis, provides a tumor with oxygen and nutrients [4,5]. Several different growth factors and cytokines drive angiogenesis such as VEGF, a predominant pro-angiogenic factor in human cancer [6,7]. Conventionally, stimulated VEGF bind to VEGF receptor 2 (VEGFR2) in tumors, contributing to the proliferation, migration and invasion of breast cancer cells. On ligand interaction, VEGFR2 is activated through receptor dimerization and autophosphorylation of tyrosine residues (Y951, Y1175, and Y1214) in its cytoplasmic kinase domain. VEGF expression may be conducive to the aggressive phenotype seen in HER2-positive breast cancer. However, VEGF is also expressed in a considerable number of HER2-negative tumors, suggesting that its expression is regulated by additional processes in breast cancer. VEGF and VEGFR2 are co-expressed in several epithelial tumors, including breast cancer, which provides further evidence for an autocrine pathway for this ligand and its receptor [8]. A relatively high cytosolic level of VEGF in breast cancer cells has been associated with the clinical aggressiveness and relapse of the cancer [9]. However, TAM is also known to increase the expression of vascular endothelial growth factor (VEGF), which is an undesirable effect in breast cancer treatment [10,11]. TAM can exert estrogen-like agonistic effects, such as induction of VEGF mRNA expression in MCF7 breast cancer cells [12-14]. Specifically, VEGF is one of the gene induced by both TAM and estrogen in rat uterine cells [15]. An elevated cytosolic level of the ligand VEGF has been associated with inferior outcome in non-randomized trials of TAM-treated hormone-responsive patients, indicating that VEGF can be a marker of response for endocrine therapy [16]. VEGF is a predictor of TAM response among ER-positive patients with either a low or high fraction of ER-positive cells [14]. VEGFR2 is an additional predictor of TAM response, with a more notable effect in ER-positive tumors. The expression levels of VEGFR2 and VEGF affect the efficacy of TAM in breast cancer patients [8]. Furthermore, adjuvant TAM administration results in shorter survival of breast cancer patients who have higher expression levels of VEGF or VEGFR2 [16]. From the above reports, we interpret that reduction in TAM dose can decrease the VEGF production. This reduction in TAM dose can be achieved by employing combination therapy.

The combination of TAM and an anti-VEGF signaling agent inhibits both ER-mediated signaling and VEGF-stimulated stromal activation, thereby reducing angiogenesis [8,17]. Studies have so far indicated that, in human breast cancers, COX-2 overexpression is correlated with induction of VEGF expression and therefore tumor angiogenesis [18]. Inhibition of COX-2 by non-steroidal anti-inflammatory drugs leads to restricted angiogenesis and down-regulates production of VEGF [19]. In pancreatic cancer, celecoxib (CXB), a selective COX-2 inhibitor, suppresses VEGF gene expression by targeting the VEGF promoter responsible for its inhibitory effect [20]. In this context, for this study we had selected CXB as non-steroidal anti-inflammatory drug in combination with TAM for suppressing VEGF expression and simultaneously reducing doses of both the drugs.

The objective of the current study was to evaluate the potency of CXB in combination with TAM in inhibiting breast cancer cell growth, proliferation, and angiogenesis and reveal the underlying molecular mechanisms involved in TAM-induced apoptosis. We also determined whether CXB, as an adjuvant agent, could reduce the dosage of TAM and its consequences in potentially reducing VEGF- and VEGFR2-mediated insensitivity in breast cancer cells to TAM.

## Methods

### Cell Lines

Human breast cancer cell lines MCF7, MDA-MB-231, MDA-MB-468, T-47D, and normal cell lines NIH/3T3 and HaCaT were obtained from the National Centre for Cell Science (Pune, India) and cultured. Cells were incubated at 37°C in a 5% CO_2_ atmosphere and at 95% humidity.

### Reagents

Stock solutions of 10 mM TAM and 1 mM CXB (Sigma Aldrich, St. Louis, MO, USA) were dissolved in dimethyl sulfoxide (Sigma Aldrich, St. Louis, MO, USA), stored at −20°C, and diluted in fresh medium just before use. For western blot analysis, the following antibodies were used: rabbit monoclonal anti-Bak, anti-CBP, anti-p-MAPK (Thr202/Tyr204), anti-MAPK, anti-p-Akt (Ser473), anti-Akt, anti-p-STAT3 (Tyr705), anti-STAT3, anti-p-Src (Tyr416), anti-Src, anti-p-VEGFR2 (Tyr1175), anti-VEGFR2, anti-p-BAD (Ser136), anti-BAD, anti-COX-2, anti-HIFα, anti-MMP-2, anti-VHL, and anti-PARP (all Cell Signalling Technology, Beverly, MA, USA), mouse monoclonal anti-β-Actin (Sigma Aldrich, St. Louis, MO, USA), and mouse monoclonal anti-Bcl2, mouse monoclonal anti-Bax, and horseradish peroxidase-conjugated goat anti-rabbit IgG and anti-mouse IgG (Santa Cruz Biotechnology, Santa Cruz, CA, USA). The pGL3-VEGFR2-780 plasmid (Addgene plasmid 21307) was kindly provided by Dr. Donald Ingber (Harvard Medical School, Boston, MA, USA), and the pGL3-Basic plasmid was purchased from Promega (Madison, WI, USA). FuGENE HD transfection reagent was purchased from Roche Applied Science (Mannheim, Germany); Opti-MEM I reduced serum medium, TRIzol reagent kit and Coomassie Blue R-250 from Gibco-BRL, Invitrogen Corporation, Carlsbad, CA, USA; Nonidet P-40 lysis buffer, chemiluminescent peroxidase substrate, propidium iodide (PI), 4′,6-diamidino-2-phenylindole (DAPI), 3-(4,5-dimethylthiazol-2-yl)-2,5 diphenyltetrazolium bromide (MTT), and sense and antisense VEGFR2 oligo primers from Sigma Aldrich, St. Louis, MO, USA; and pyrogallol and H_2_O_2_ from Merck (Whitehouse Station, NJ, USA). Stock solutions of PI, DAPI, and MTT were prepared by dissolving 1 mg of each compound in 1 ml of phosphate-buffered saline (PBS). The solution was protected from light, stored at 4°C, and used within 1 month. Stock concentrations of 10 mg/ml RNaseA (Sigma Aldrich, St. Louis, MO, USA) were prepared and kept at −20°C.

### Cell viability assay

MCF7 and MDA-MB-231 cells grown in monolayers were harvested and dispensed in 96 well culture plates in 100 μl of Dulbecco’s Modified Eagle’s Medium (DMEM) at a concentration of 5 × 10^3^ cells per well. After 24 h, differential drug concentrations of TAM (0–40 μM), CXB (0–250 μM), or both (0–5 μM TAM plus 30 μM CXB) were added to the cells. Cell viability was measured after 48 h of incubation using the MTT colorimetric assay at 540 nm with slight modifications to the protocol [21]. The dose-effect curves were analyzed using Prism software (GraphPad Prism, CA, USA).

### Cell cycle analysis

To determine the cell cycle distribution, 5 × 10^5^ MCF7 or MDA-MB-231 cells were plated in 60-mm dishes and treated with their respective half maximal inhibitory concentration (IC_50_) values of TAM, CXB, or both for 48 h. After treatment, the cells were collected by trypsinization, fixed in 70% ethanol, and kept at −20°C overnight for fixation. Cells were washed in PBS, resuspended in 1 mL of PBS containing 100 μg/mL RNase and 40 μg/mL PI incubated in the dark for 30 min at room temperature [22-24]. The distribution of cells in the cell-cycle phases were analyzed from the DNA histogram using a FACS Caliber flow cytometer (Becton-Dickinson, San Jose, CA, USA) and CellQuest software (CA, USA).

### Wound-closure assay

To assess the effect of TAM and CXB on cell migration, MCF7 and MDA-MB-231 cells (1 × 10^5^) were plated in 12-well plates in complete growth medium [23,25]. After 24 h of growth, a scratch was made through the confluent cell monolayer using a 200-μl pipette tip, and the cells were treated with the IC_50_ values of TAM, CXB, or both in 3 ml of complete medium. At 48 h post-treatment, cells were stained with hematoxylin and eosin. Cells invading the wound line were observed under an inverted phase-contrast microscope using 20×, Leica DMR, Germany. The distance between the two sides of the scratch was measured after the indicated time intervals using Leica QWin software, IL, USA. Each experiment was performed three times with triplicate samples.

### Boyden chamber assay

To test the anti-invasive effect of TAM and CXB, 8-μm filters were coated with Matrigel (20 μg per filter) and placed in Boyden chambers. MDA-MB-231 cells (1 × 10^5^) suspended in DMEM containing 0.1% bovine serum albumin and treated with IC_50_ of TAM, CXB, or both, were added to the top chamber. Conditioned medium from mouse fibroblast NIH/3T3 cells was used as a source of chemoattractant and placed in the bottom compartment of the chamber [26]. After 24 h incubation at 37°C in a 5% CO_2_ atmosphere, cells that migrated to the lower surface of filters were detected with traditional staining with hematoxylin and eosin. Cells were counted in five fields of each well under inverted phase-contrast microscope using 20×, Leica DMR, Germany.

### Gelatin zymography

Supernatants from MCF7 and MDA-MB-231 cells (5 × 10^4^ cells per well, six wells per plate) treated with TAM, CXB, or both for 48 h were collected for matrix metalloproteinase (MMP) activity analysis by sodium dodecyl sulfate-polyacrylamide gel electrophoresis under non-reducing conditions. A total of 1.2 mg/ml gelatin was prepolymerized on a 10% polyacrylamide gel as a substrate. Electrophoresis was carried out at 4°C. The gel was washed with renaturation buffer (50 mM Tris–HCl, pH 7.5, 100 mM NaCl, and 2.5% Triton X-100), which was followed by incubation with a developing buffer (50 mM Tris–HCl, pH 7.5, 150 mM NaCl, 10 mM CaCl_2_, 0.02% NaN_3_, and 1 μM ZnCl_2_) at 37°C for 16 h and staining with Coomassie Blue R-250, as described previously [27]. The stained bands are observed through a gel doc system (Bio-Rad). Densitometric analysis of stained bands was performed by ImageMaster 2D Platinum 7.0 Software (GE Healthcare Life Sciences, NJ, USA).

### Chorioallantoic Membrane (CAM) assay

To determine the in vivo anti-angiogenic activity of TAM and CXB, a CAM assay was performed as described previously with some modifications [28]. Two day-old fertilized eggs were incubated at 37°C in 60–70% relative humidity. After 5 d of incubation, a 1- to 2-cm^2^ window was opened and a sterile round filter paper (5-mm in diameter; Whatman qualitative filter papers, Sigma-Aldrich, St. Louis, MO, USA) containing serum-free medium alone or supplemented with VEGF, TAM, CXB, or both TAM and CXB (at IC_50_ concentrations) was applied onto the CAM of each embryo. After 2 d of incubation, the upper eggshell was removed, and capillaries within 2.5 mm around the filter paper were observed and photographed under a stereomicroscope (Olympus, SZX16, USA). Neovascularization around the disk was quantitated by determining the number of angiogenic vessels within the CAM around the disk.

### Capillary-like tube formation (HUVEC) assay

For the capillary-like tube formation assay, growth factor-depleted Matrigel from BD Pharmingen, San Jose, CA, USA was applied to a 96-well tissue culture plate (50 μl per well). After polymerization of the Matrigel at 37°C for 1 h, human umbilical vein endothelial cells (HUVECs) (Gibco-BRL, Invitrogen Corporation, Carlsbad, CA, USA) starved of serum for 2 h were harvested by using trypsin/EDTA, washed with assay medium, and seeded at a density of 7.5 × 10^3^ cells per well (final volume 500 μl) on the polymerized Matrigel in the presence or absence of 30 ng/ml VEGF along with TAM, CXB, or both [29,30]. Plate was incubated at 37°C, 5% CO_2_ for 24 h, then the medium was aspirated and cells were fixed in 10% neutral buffered formalin. Tube formation was observed for 24 h, representative pictures were taken at 10× magnifications under a stereomicroscope (Olympus, SZX16, USA) and tubes were counted in five random fields.

### Western blotting analysis

For phosphoprotein studies, MCF7 and MDA-MB-231 cells (1 × 10^6^ cells per 100 mm plate) were treated with TAM, CXB, or both at their respective IC_50_ doses for 24 h. Cells in control wells were treated with 0.1% dimethyl sulfoxide for 1 h. All cells were activated with recombinant human epidermal growth factor (25 ng/mL) for 30 min. The cells were then scraped and lysed in Nonidet P-40 lysis buffer. Cell extracts (50 μg of protein) were separated on a sodium dodecyl sulfate-polyacrylamide electrophoretic gel and transferred to nitrocellulose membranes, which were blocked in 3% bovine serum albumin for 2 h. After blocking, the membranes were incubated with primary antibodies overnight at 4°C and then with horseradish peroxidase-conjugated secondary antibody for 2 h at room temperature [24]. Proteins were visualized by exposing the chemiluminescence substrate (Sigma) to X-OMAT AR autoradiography film (Eastman Kodak, Rochester, NY, USA).

### Transfection studies

MCF7 and MDA-MB-231 cells were plated in 60-mm petri dishes at a density of more than 4 × 10^5^ per plate in DMEM supplemented with 10% fetal bovine serum. After being allowed to grow for 16–20 h, cells were starved for 6 h with 2% fetal bovine serum. Confluent cells (70–80%) were transiently transfected with 5 μg of pGL3-VEGFR2-780 plasmid with 7.5 μl of FuGENE HD transfection reagent in 100 μl of Opti-MEM I reduced serum medium according to the manufacturer’s protocol (Roche Diagnostics, Mannheim, Germany) [31]. After 24 h of transfection, the mix was replaced with complete medium containing TAM, CXB, both, or neither for 24 h and then lysed in luciferase lysis buffer (Sigma) [32,33]. Luciferase activity was measured with a luminometer (Varian cary eclipse, Palo Alto, CA, USA) and a luciferase assay kit (Sigma) and was normalized to β-galactosidase activity. All luciferase experiments were done in triplicate and repeated three times. Data is presented as means ± SD.

### Measurement of VEGF levels

To measure VEGF levels, MCF7 and MDA-MB-231 cells (5 × 10^5^ cells per well, six wells per plate) were plated and incubated under culture conditions overnight, and the medium was replaced by serum-free culture conditioned medium. TAM, CXB, or both were added to the culture, and the medium was collected at 72 h [10]. VEGF levels were measured using a VEGF enzyme-linked immunosorbent assay (ELISA) kit (DVE00, R&D Systems, Minneapolis, MN, USA) according to the manufacturer’s instructions. The optical density at 570 nm of each well was measured using an automated microplate reader (model 550, Bio-Rad, Hercules, CA, USA).

### Reverse transcription-polymerase chain reaction (RT-PCR)

By using the TRIzol reagent kit, total RNA was extracted from MCF7 and MDA-MB-231 cells treated with TAM, CXB, or both. RT-PCR was run using a one-step RT-PCR kit (Gibco-BRL, Invitrogen Corporation, Carlsbad, CA, USA). β-Actin was used as an internal control. The sense and antisense primers for the VEGFR2 gene were 5′-TGACCAACATGGAGTCGTG-3′ and 5′-CCAGAGATTCCATGCCACTT-3′, respectively. The sense and antisense primers for β-Actin were 5′-TCATGTTTGAGACCTTCAA-3′ and 5′-TCTTTGCGGATGTCCACG-3′, respectively. PCR was performed in a 25-μL reaction volume. The cycling conditions were 94°C for 5 min; 35 cycles of 94°C for 30 s, 54°C for 45 s, and 72°C for 60 s; and a final extension at 72°C for 10 min. Amplified products were separated by 1.2% ethidium bromide-stained agarose gel electrophoresis and viewed under ultraviolet light. Electrophoresis photos were transferred to a computer and analyzed using the Gel Doc image system (Bio-Rad) [34]. Semiquantitative analysis was performed by comparing the results of VEGFR2 mRNA with β-Actin.

### Animal studies

Tumor response to CXB and TAM was studied using S180 tumor bearing female Swiss albino mouse model. Our study was approved by the Department of Biotechnology (DBT), INDIA under the project number: E-1/MMSMST/12, at Indian Institute of Technology Kharagpur, INDIA and the mice were maintained in accordance with the institute animal ethical committee (IAEC) guidelines approved by Indian Council of Medical Research (ICMR), New Delhi. The mice were housed and acclimatized in a pathogen-free environment at our institute’s animal facility for 1 week prior to injection with mouse S180 sarcoma cells. Exponentially growing S180 cells were harvested and a tumorigenic dose of 2.5 × 10^6^ cells was injected intraperitoneally into 6- to 7-week-old female Swiss albino mouse [24,35,36]. Tumors were allowed to grow in the mouse for 7 d, when the animals were randomly assigned into one of four treatment groups (5 mice per group). The control group received 1% polysorbate resuspended in deionized water. The other three groups were treated with CXB (3.7 mg/kg body weight), TAM (2 mg/kg body weight), or CXB plus TAM (2 and 1 mg/kg body weight, respectively) intraperitoneally on alternative days for 2 weeks. The doses were selected based on previous experiments [37,38]. Mouse body weight was measured before the treatment injections were given and on the 7^th^ and 14^th^ day of treatment. On 15^th^ day, the animals were euthanized using chloroform and their liver and kidney tissues were collected for enzymatic assays. Spleens were collected and cultured for a splenocyte surveillance study. Furthermore, S180 cells were collected from the site of treatment injections for in vivo and ex vivo cell cycle phase distribution studies.

### Assay of splenocyte proliferation

Spleens from treated mice were collected, and single-cell spleen suspensions were pooled in serum-free DMEM by filtering the suspension through a sieve mesh with the aid of a glass homogenizer to exert gentle pressure on the spleen fragments. Samples were washed twice in PBS 0.1% (w/v) bovine serum albumin. After centrifugation at 200 *g* for 5 min, the cells were placed into 96-well flat-bottomed microplates in triplicate at 2.5 × 10^3^ cells per well in DMEM supplemented with 10% fetal bovine serum. The cells were then incubated in a total volume of 100 μL per well. Serum-free DMEM was used as control [39]. After 24 h, cell proliferation was measured using the MTT assay.

### Measurement of antioxidative enzyme activity

Parts of mouse liver and kidney tissues were homogenized in 0.1 M Tris buffer (pH 7.0), and the homogenate was centrifuged at 4000 *g* for 20 min. The supernatant was immediately assayed for catalase (CAT) and superoxide dismutase (SOD). Determination of CAT activity was performed at room temperature in a 1-ml mixture containing clear cell lysate, 100 mM phosphate buffer (pH 7.0), and 10 mM of H_2_O_2_[40]. The decomposition of H_2_O_2_ is followed directly by a decrease in absorbance at 240 nm spectrophotometrically using Perkin Elmer Lambda45. CAT activity was expressed in micromoles of H_2_O_2_ consumed per minute per milligram of protein.

Total SOD was determined using the pyrogallol assay, based on the competition between pyrogallol oxidation by superoxide radicals and superoxide dismutation by SOD [41], and spectrophotometrically read at 420 nm using Perkin Elmer Lambda45. SOD activity was expressed in units per minute per milligram of protein.

### Measurement of ROS

To measure intracellular reactive oxygen species (ROS), 10 μM 2′,7′-dichlorofluorescein diacetate (DCFDA) was used [28]. MCF7 and MDA-MB-231 (5 × 10^4^ cells per well, six wells per plate) were treated with IC_50_ of TAM, CXB, or both for 24 h; washed with PBS; stained with DCFDA at a final concentration of 1 μg/ml for 30 min at 37°C; and subjected to flow cytometry (FACS Calibur flow cytometer, Becton-Dickinson). Data were acquired and analyzed with CellQuest software.

### Statistical analysis

All the statistical analysis was performed by Graphpad Prism 5 software. Data are presented using mean ± S.D. The statistical significance was determined by using one-way analysis of variance (ANOVA). ***P < 0.001 and **P < 0.05 were considered significant.

## Results

### CXB enhances TAM-induced breast cancer cell death

**Figure 1:**
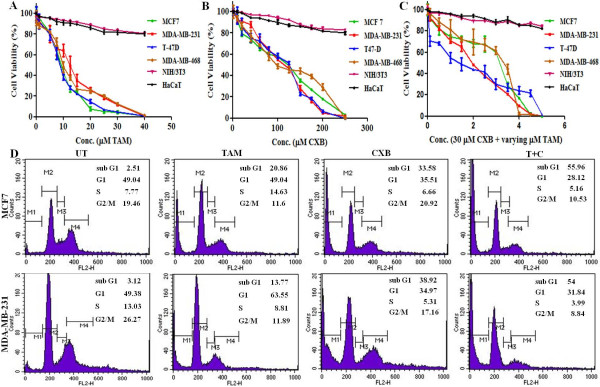
**TAM combined with CXB additively inhibits survival of breast cancer cells.** In vitro cell viability assay of MCF7, MDA-MB-231, T-47D, MDA-MB-468, NIH/3T3 and HaCaT cells treated with (**A**) TAM, (**B**) CXB, or (**C**) both (0–5 μM TAM plus 30 μM CXB) for 48 h. Data are means ± SE of three independent experiments p < 0.05. (**D**) Representative histogram of MCF7 cells (top row) and MDA-MB-231 (bottom row) cells and their cell-cycle distribution after 48 h of treatment, as determined by flow cytometry followed by staining of cells with PI. T + C, TAM plus CXB; UT, untreated.

To determine the effect of TAM, CXB, and both on the cell viability of breast cancer cells in vitro, ER-α-positive MCF7 and T-47D cells and ER-α-negative MDA-MB-231 and MDA-MB-468 cells were treated with increasing concentrations of CXB (0–250 μM) or TAM (0–40 μM). Treatment with TAM alone resulted in similar IC_50_ values for the MCF7, T-47D, MDA-MB-231, and MDA-MB-468 cell lines (9.06 ± 0.29, 8.99 ± 0.55, 13.05 ± 0.91, and 11.56 ± 0.65 μM, respectively) (Figure 1A). Treatment with CXB alone also resulted in IC_50_ values that were similar in these four cell lines (113.3 ± 0.760, 109.3 ± 0.782, 109.8 ± 0.963, and 121.7 ± 0.240, respectively) (Figure 1B). Combination treatment (0–5 μM TAM in the presence of 30 μM CXB) resulted in a leftward shift of the concentration-response curve such that the IC_50_ values were reduced to 2.76 ± 0.10, 1.82 ± 0.13, 2.05 ± 0.13, and 2.86 ± 0.12 μM, respectively (Figure 1C), indicating that treatment with both agents was more cytotoxic than either one alone. The treatment regimens resulted in little toxicity in NIH/3T3 and HaCaT cell lines, demonstrating that TAM and CXB are non toxic to normal cell lines. Based on the results we have chosen respective IC_50_’s of drugs for further treatments throughout the study.

### CXB enhances TAM-induced apoptosis and growth inhibition

The effects of TAM and CXB on the cell cycles of MCF7 and MDA-MB-231 cells were then analyzed. MCF7 cells (IC_50_ values: 114 μM CXB, 9 μM TAM) treated with TAM or CXB had an increased percentage of apoptotic cells (i.e., cells in the sub-G_1_ phase) compared with untreated cells (Figure 1D, top row). Similarly, MDA-MB-231 cells (IC_50_ values: 110 μM CXB, 13 μM TAM) had an increased percentage of apoptotic cells compared with untreated cells (Figure 1D, bottom row). The low-dose combination (30 μM CXB plus 2 μM TAM) resulted in an even greater percentage of apoptotic cells than the higher doses of either drug alone did. These data are consistent with the results from the MTT assay. Taken together, these results indicate an additive mechanism of TAM and CXB in inducing cell death through apoptosis.

### Effect of TAM and CXB on migration and invasion of breast cancer cells

**Figure 2:**
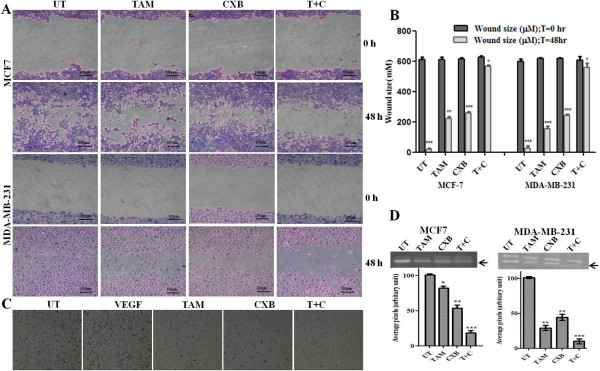
**Anti-invasive and anti-migratory potential of TAM and CXB in MCF7 and MDA-MB-231 cells.** (**A**) Representative hematoxylin- and eosin-stained cell images migrating into the wounded area in an in vitro wound healing assay at times 0 h and 48 h. Scale bars, 100 μm. (**B**) Quantification of wound-healing results. Data are means ± SE of three random widths along the wound. P < 0.05. (**C**) Representative photomicrographs of Boyden chamber assays of MDA-MB-231 cell invasion through Matrigel. Cells were stained with hematoxylin- and eosin. (**D**) Top: Gelatinolytic activity of MMP-2 in MCF-7 cells and MDA-MB-231 cells treated for 48 h. Bottom: Densitometric analysis of MMP-2 protein levels in gelatin blot. Data are means ± SE of three independent experiments. P < 0.05 (*t*-test).T + C, TAM plus CXB; UT, untreated cells.

To ascertain the inhibitory effect of TAM and CXB on breast cancer metastasis, we used the wound-healing assay to investigate their effects on the migration potential of MCF7 and MDA-MB-231 cells. A wound through a confluent cell monolayer was created with a pipette tip, and the migration of cells to fill up the wound was recorded by microscopic observation. After 48 h, the wound had almost completely filled in the cleared region in untreated MCF7 and MDA-MB-231 cells (Figures 2A and 2B). The migration of MDA-MB-231 cells was reduced with TAM or CXB with respect to the untreated cells and greatly reduced when both TAM and CXB were used. However, TAM and CXB had limited effects in MCF7 cells, which might be explained by the poor invasiveness of this cell line.

The ability of TAM and CXB to reduce the invasiveness of MDA-MB-231 cells was further investigated by the Boyden chamber assay. Cells treated with IC_50_ concentrations of TAM, CXB, or both for 24 h were plated in the upper chamber, and the number of cells that moved to the underside of the coated membrane was counted 12 h later using a light microscope. The chambers were stained with hematoxylin and eosin and analyzed by photography. Again, compared with the results with either agent alone, the combination of TAM and CXB greatly inhibited MDA-MB-231 cell invasion (Figure 2C).

### TAM and CXB inhibit activation of MMP-2 in breast cancer cell lines

Substantial levels of MMP secretion have been reported for metastatic breast cancer tumors and to be associated with the degradation of extraceullular matrix, a crucial step in metastasis [42]. Zymographic analyses showed that TAM and CXB additively inhibited MMP-2 activity in both MCF7 and MDA-MB-231 cells (Figure 2D). Thus, apart from its anti-VEGF effect in inhibiting tumor cells, this combination treatment can inhibit the metastasis and spread of breast cancer cells by reducing MMP-2. The addition of CXB enhanced the anti-metastatic potential by more than 2-fold in comparison to control. However, the impact of TAM and CXB on MMP-9 activity is inconclusive because an extremely low level of MMP-9 was detected in untreated cells (data not shown).

### TAM and CXB inhibit in vivo angiogenesis and in vitro tube-like capillary formation

**Figure 3:**
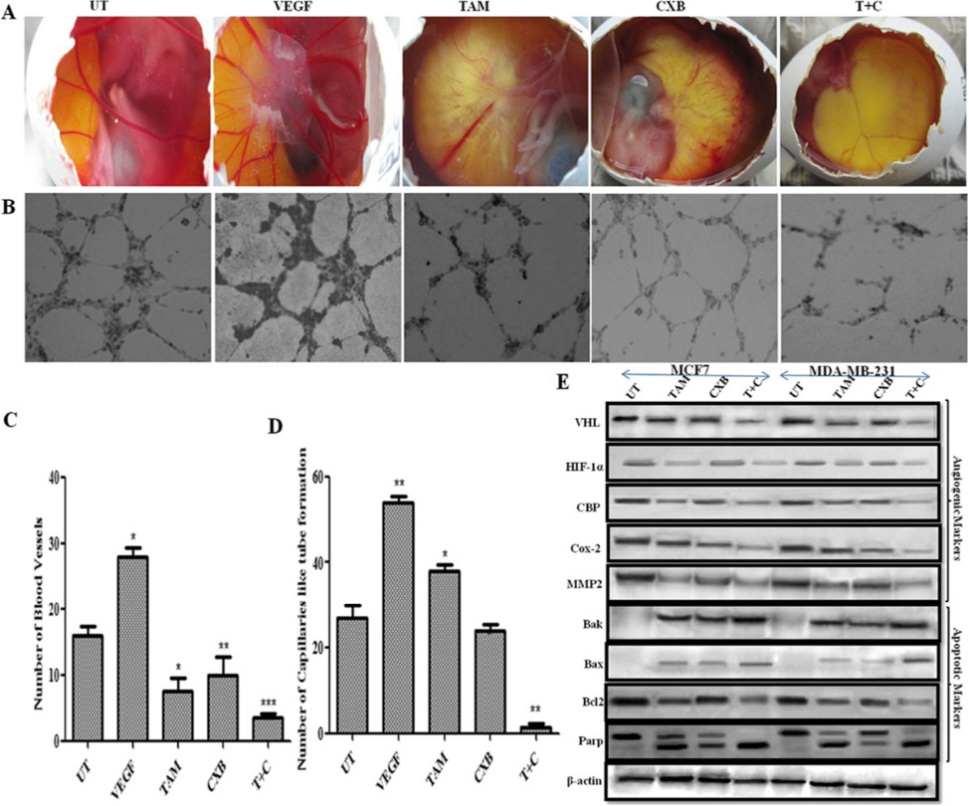
**Anti-angiogenic and anti-tube formation potential of TAM and CXB.** (**A**) In vivo CAM assay. CAMs were implanted with sponges loaded with serum-free medium alone or supplemented with vascular endothelial growth factor (VEGF), TAM, CXB, or TAM plus CXB. (**B**) Inhibition of capillary-like tube formation in vitro (HUVECs assay). HUVECs were seeded (7.5 x 10^3^ cells/well) into a 96-well tissue culture plate coated with 50 μl Matrigel. Then, TAM and/or CXB were added. Cells were incubated in HUVEC growth medium in a 37°C, 5% CO_2_ incubator. Tube formation was observed for 24 h and images were taken (magnification of 10×). (**C**) Number of blood vessels in CAM assay was counted as means ± SD of blood vessel count for four independent experiments P < 0.05. (**D**) Number of capillary-like structures in capillary-like tube formation assay was counted using light microscopy. Data are presented as means ± SD of four independent experiments. (**E**) Western blotting analysis of apoptotic and angiogenic markers in MCF7 and MDA-MB-231 cells treated with TAM, CXB, or both. β-Actin was used as an invariant control for equal loading. Representative blots from three independent experiments are shown. T + C, TAM plus CXB; UT, untreated cells.

The CAM model was used to investigate the effect of TAM and CXB on angiogenesis in vivo [43]. CAM assay with the PBS group did not show any notable avascular zone around the implanted filter paper (Figures 3A and 3C). In contrast, treatment with TAM, CXB, and both agents together inhibited the development of new embryonic capillaries and produced an avascular zone around the implanted filter papers. The inhibition of angiogenesis was most prominent when TAM and CXB were combined.

Next we performed tube formation assays with HUVECs, which are widely used as in vitro assays for angiogenesis. After 24 h, HUVECs treated with PBS only rapidly aligned and formed hollow, tube-like structures, whereas HUVECs treated with both TAM and CXB showed a significant reduction of tube formation compared with TAM or CXB alone (Figures 3B and 3D). Collectively, these results suggest that CXB enhances the anti-angiogenic action of TAM by inhibiting HUVEC differentiation into tube-like structures during angiogenesis.

### TAM and CXB inhibit angiogenesis via von Hippel-Lindau tumor suppressor protein (VHL)-mediated degradation of hypoxia-inducible factor 1α (HIF-1α)

VHL regulates activated HIF-1α through ubiqitination by prolyl hydroxylation under normoxia conditions [44]. In reduced oxygen conditions, HIF-1α binds to hypoxia-responsive elements which, in turn, stimulate the transcriptional coactivators CREB-binding protein and induces transcription of various target genes involved in tumor invasion, cell survival, and angiogenesis. Apart from its role in angiogenesis, HIF-1α promotes invasion by regulating the expression of COX-2, MMP-2, and other cytokines and growth factors [45]. Our western blotting results demonstrated that the combination of TAM and CXB modulated VHL expression in MCF7 and MDA-MB-231 cells, thus regulating HIF-1α, which in turn binds to CREB-binding protein, thereby altering the expression of the downstream effector molecules involved in metastasis and angiogenesis (e.g., MMP-2, COX-2 and VEGF) (Figure 3E). These features have rendered HIF-1α as an attractive target for our study in inhibiting angiogenesis.

### TAM plus CXB lowers VEGF production in breast cancer cells

**Figure 4:**
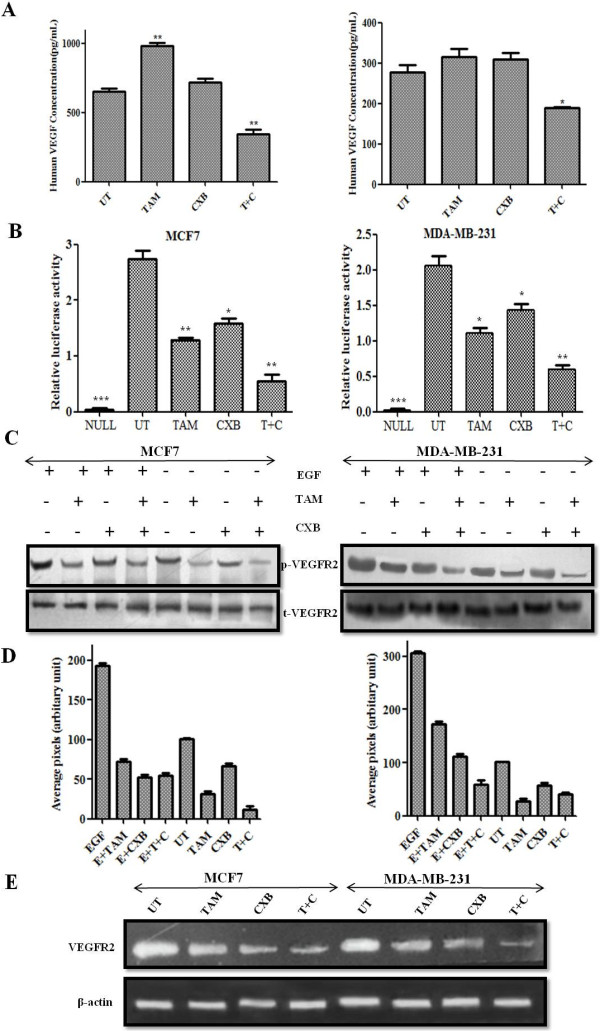
**TAM- and CXB- inhibit overexpressed VEGFR2 induced angiogenesis in MCF7 cells (left) and MDA-MB-231 cells (right).** (**A**) Cells were treated with TAM, CXB, or both and incubated in serum-free conditioned medium for 24 h. VEGF levels were determined by ELISA. (**B**) Cells (5 × 10^5^/ml) were transfected with VEGFR-luciferase plasmid, incubated for 24 h, and treated with TAM, CXB, or both for 4 h. Whole-cell extracts were then prepared and analyzed for luciferase activity. Absolute values are normalized to untreated cells without VEGFR2. Data are means ± SD of three independent experiments. (**C**) Western blot analysis for VEGFR2 and phosphorylated VEGFR2. (**D**) Densitometric analysis of phosphorylated VEGFR2 protein levels. Data are means ± SD of three independent experiments. P < 0.05 (*t*-test). (**E**) The level of VEGFR2 mRNA in MCF7 and MDA-MB-231 cells examined by RT-PCR analysis following TAM, CXB and T + C treatment for 24 h. Data are means ± SD of three independent experiments using different cell preparations. *P < 0.05 vs. untreated cells. EGF, epidermal growth factor; T + C, TAM plus CXB; UT, untreated cells.

We investigated the role of TAM and CXB in the inhibition of secretory VEGF, a pro-angiogenic factor responsible for the migration and invasion of breast cancer cells. VEGF secretion in serum-free culture conditioned medium was assessed in MCF7 and MDA-MB-231 cells by ELISA 24 h post-treatment. In both cell lines, TAM alone considerably upregulated VEGF secretion and the combination of CXB and TAM notably decreased VEGF secretion compared with no treatment (Figure 4A). Precisely, in control cells VEGF levels were found to be approximately 600 and 280 pg/mL in MCF7 and MDA-MB-231 cells, respectively whereas CXB treatment alone does not showed any significant change in the secreted VEGF levels in both cell lines. However, induced VEGF was suppressed in combination treatment to 400 pg/mL in MCF7 and 190 pg/mL in MDA-MB-231 in comparison to TAM alone treated MCF7 (1000 pg/mL) and MDA-MB-231 (320 pg/mL).

### TAM plus CXB inhibits VEGF-mediated stimulation of VEGFR2 promoter activity

To further confirm the role of enhanced activity induced by treatment with TAM and CXB in the transcriptional regulation of the *VEGFR2* gene, cells were transiently transfected with a chimeric luciferase gene fused with the 5′ region of the VEGFR2 promoter (Tischer et al., 1991), and the activity of the promoter was assayed in the presence and absence of *VEGFR2* gene after treatment with the IC_50_ doses for 24 h. Transfection induced VEGFR2 promoter activity in both MCF7 and MDA-MB-231 cells. To determine the relative fold change in VEGFR2 promoter activity, we normalized with respect to untransfected control (null) cells. VEGFR2 transfected untreated cell (UT) showed an approximately 3- and 2-fold increase in promoter activity as compare to null in MCF7 and MDA-MB-231 cells, respectively. There was an approximately 1.2-fold increase in VEGFR2 promoter activity in TAM-treated and approximately 1.5-fold increase in CXB-treated whereas fold increase was observed <1 in TAM-CXB treated with respect to null in both cell lines. Concisely, TAM and CXB was effective in blocking VEGFR2 promoter induced expression in MCF7 and MDA-MB-231 cells (Figure 4B). Taken together, the results of this experiment demonstrated that the activity of the VEGFR2 promoter is downregulated by CXB under the influence of TAM in both the cell lines. Besides, it also interferes with the phosphorylation of VEGFR2 (Figures 4C and 4D). Further, RT-PCR analysis was also in accordance with the VEGFR2 promoter luciferase activity (Figure 4E).

### TAM and CXB in combination suppress VEGFR2-mediated Src/STAT3/Akt/MAPK signaling

**Figure 5:**
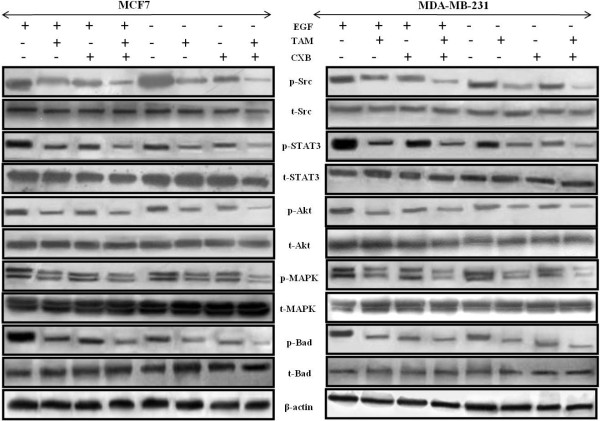
**Phosphoprotein and total protein expression profiles of MCF7 (left) and MDA-MB-231 (right) breast cancer cells treated with TAM and/or CXB.** Phosphorylated levels of p-Src (Tyr416), p-STAT3 (Tyr705), p-Akt (Ser473), p-MAPK (Thr202/Tyr204) and p-Bad (Ser136) were determined by western blot analysis using their specific antibodies. β-Actin was used as an invariant control for equal loading.

VEGFR2 is the major receptor of VEGF in angiogenesis, and the VEGF/VEGFR2 pathway plays a central role in angiogenesis. TAM and CXB together strongly inhibited VEGF-activated VEGFR2 phosphorylation at Tyr1175 in western blotting analysis of MCF7 and MDA-MB-231 cells (Figures 4C and 4D). To determine whether this combination treatment could inhibit downstream signaling of VEGFR2, we screened some key factors involved in the VEGFR2 signaling pathway. Here, EGF was employed as a growth stimulant to induce phosphorylation levels of regulatory proteins. For both cell lines, the phosphorylation activities of Src and STAT3 were much lower with the combination than with either drug alone (Figures 4C, 4D and Figure 5). Because STAT3 plays an important role as a critical transcription activator in angiogenesis, we then analyzed the expression of STAT3 downstream genes. Results showed that compared with TAM or CXB alone, TAM-CXB together inhibited the expression of anti-apoptotic Bcl-2 protein and increased the levels of pro-apoptotic Bax and Bak proteins (Figure 3E). STAT3 is also involved in the inhibition of apoptosis in endothelial cells. We found that various death substrates, such as poly(ADP-ribose) polymerase (PARP) (Figure 3E) and other molecules at conserved aspartic acid residues (data not shown), were more strongly activated by TAM-CXB in combination than by either drug alone in MCF7 and MDA-MB-231 cells. Taken together, these western blotting analysis results suggest that the combination of TAM-CXB blocks the VEGF-induced Src/STAT3 signaling pathway. Further, our western blotting analysis proved the involvement of VEGFR2 signaling in the inhibition of AKT and MAPK and the phosphorylation of the downstream protein Bad (Figure 5). Bad plays important roles in tumor cell function, angiogenesis, and tumor growth.

### TAM plus CXB causes significant inhibition of S180 tumors

**Figure 6:**
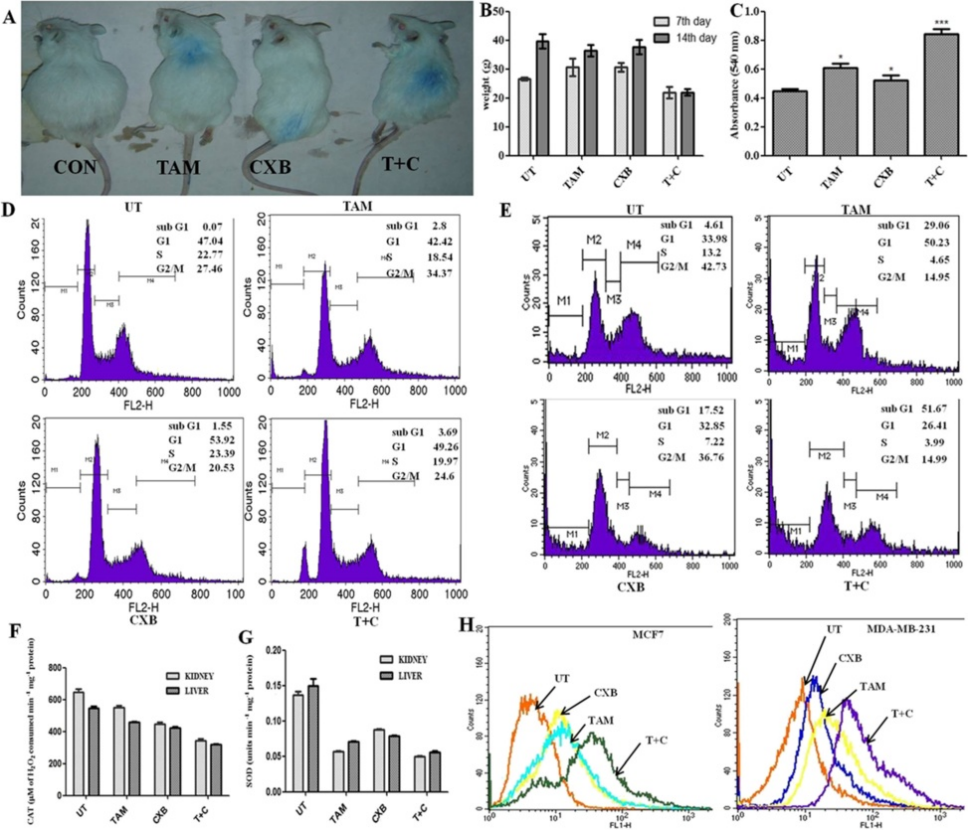
**Antitumor activity of TAM and CXB in Swiss albino mice bearing S180 tumors.** Mice were intraperitoneally injected with TAM (2 mg/kg body weight), CXB (3.7 mg/kg body weight), or both (2 and 1 mg/kg body weight, respectively) on alternative days after tumor cell implantation and continued for 2 weeks. (**A**) Mice images bearing S180 tumors with different treated groups at the time of sacrifice, (**B**) Animal body weight on 7th and 14^th^ day of treatment. Data are means ± SD of three independent experiments. (**C**) MTT assay of proliferation of splenocytes from mice. Data are means ± SD of three independent experiments. p < 0.05 compared with untreated mice. (**D**) Cell-cycle phase distribution study of S180 cancer cells isolated from the intraperitoneal region of treated animals exposed to TAM and CXB for 48 h followed by PI staining. (**E**) Cell-cycle phase distribution analysis of ex vivo grown S180 cells exposed to TAM and CXB for 48 h followed by PI staining. Enzyme activity assays of catalase (**F**) and superoxide dismutase (**G**) from liver and kidney tissue homogenates of S180 tumor-bearing Swiss albino mice after drug treatment. Data are means ± SD of three independent experiments. (**H**) Intracellular ROS accumulation in MCF-7 cells (top) and MDA-MB-231 cells (bottom) treated with TAM, CXB, or both for 24 h was assessed by DCFDA staining and performed flow cytometry. T + C, TAM plus CXB; UT, untreated cells.

We assessed the in vivo therapeutic efficacy of TAM and CXB in Swiss albino mice bearing S180 tumors. TAM and CXB each induced tumor regression and slowed tumor growth in these mice treatment groups (Figure 6A). Body weight of the animals was measured during the 7^th^ and 14^th^ day of treatment. Untreated mice and mice treated with TAM or with CXB gained weight over time; in contrast, whereas mice treated with both TAM and CXB maintained their weight (Figure 6B).

### CXB increases TAM-induced Splenocyte proliferation

To assess the efficacy of TAM and CXB in modulating splenocyte proliferation, spleen cells of treated S180 mice were isolated and cultured in DMEM supplemented with 10% fetal bovine serum for 24 h and subjected to in vitro proliferation assays. Compared with untreated mice, mice treated with TAM, CXB, or both displayed approximately 1.5-, 1.2-, and 2.0-fold increases, respectively, in splenocyte proliferation (Figure 6C).

### Apoptotic effects of TAM and CXB on S180 tumor cells

To assess the therapeutic efficacy of TAM and CXB, cells isolated from the intraperitoneally injected region of sacrificed mice were subjected to cell cycle analysis. Imprints of cytotoxic effects of these drugs were found at this region. The proportion of cells from untreated mice or mice treated with TAM, CXB, or both agents that was in the sub-G_1_ phase was 0.07 ± 0.56%, 2.8 ± 0.16%, 1.55 ± 0.84%, and 3.69 ± 0.63%, respectively (Figure 6D). Ex vivo cell cycle studies showed analogous results (4.61 ± 0.27%, 29.06 ± 0.13%, 17.52 ± 0.77%, and 51.67 ± 0.34%, respectively), to that of in vitro studies as shown in (Figure 6E) thereby confirming the additive therapeutic effect of the drugs.

### TAM and CXB additively decrease CAT and SOD activity

CAT and SOD assays were performed to assess the role of reactive oxygen species in VEGF induction [46]. The activities of the antioxidant enzymes CAT and SOD in the liver and kidney of S180 tumor-bearing mice were assayed. For both TAM- and CXB-treated mice, the levels of CAT activity in liver tissue or in kidney tissue were significantly lower than those of untreated mice (Figure 6F). In addition, for mice treated with both TAM and CXB, CAT activity in liver or kidney tissue was significantly lower than that in mice treated with TAM or CXB alone. Similar results were observed with SOD activity (Figure 6G).

### Role of ROS in the combined effect of TAM and CXB

To establish whether treatment with TAM and CXB for 24 h induces ROS-dependent apoptosis, we investigated whether they increase ROS generation in MCF7 and MDA-MB-231 cells by measuring the intracellular levels of H_2_O_2_ using DCFDA staining. Flow cytometric analysis revealed that for both cell lines, TAM resulted in higher generation of ROS than CXB (Figure 6H). In addition, treatment with both agents increased ROS production by over 50% as compared with the control cells, which was associated with enhanced apoptosis.

## Discussion

TAM has been described as ‘the most important drug developed in the history of breast cancer’ [47]. The introduction of TAM heralded a new approach to the treatment of breast cancer. Initial clinical studies of TAM displayed its antiangiogenic and VEGF reducing ability in various tumor models [5,48-51]. Despite its meritorious stand in the treatment of breast cancer, prolonged administration of TAM causes intracellular VEGF levels to rise in patients, an undesirable response leading to enhanced metastasis and angiogenesis and resulting in inferior outcomes [14,52]. In addition, autocrine VEGF/VEGFR2 loop activation confers resistance to TAM in breast cancer cells [8]. In this perspective, we made an attempt to decrease intracellular VEGF levels by reducing the TAM dose in ER-positive and ER-negative breast cancer cells. For accomplishing the above goal we employed combination therapy by decreasing TAM dose and choose CXB, a selective COX-2 inhibitor as an adjuvant agent [53] that induces apoptosis through inhibiting angiogenesis by suppressing VEGF expression in gastric and breast cancers [20,54]. From the above report, in the current study we aimed to determine the expression profile of VEGFR2 and quantify VEGF in both MCF7 and MDA-MB-231 breast cancer cells treated with TAM, CXB or both. In our study, we observed reduction in VEGF levels in TAM and CXB treated MCF7 but no significant change in MDA-MB-231. Interestingly, we also found that the activity of VEGFR2 was inhibited by TAM and CXB in very low concentrations than either drug alone.

STAT proteins comprise a transcription factor family that participates in normal cellular events, such as proliferation, apoptosis and angiogenesis [55]. An increasing amount of evidence has suggested that STATs, mainly STAT3, play a critical role in angiogenesis. Indeed, activated STAT3 is a mediator and biomarker of VEGF-induced endothelial activation [56]. The VEGF/VEGFR2-mediated STAT3 signaling pathway is a potential key target of anti-angiogenic tumor therapy [57,58]. Here, we elucidated the VEGFR2-activated STAT3 signaling pathway in human breast cancer cells. In our study, the activity of VEGFR2 was more strongly inhibited, and thus the activation of Src and STAT3 is suppressed by the combination of CXB and TAM (in very low concentrations) than either drug alone. The reduction of STAT3 activation, in turn, inhibited the downstream gene expression of the anti-apoptotic Bcl-2 protein and increased the expression levels of the pro-apoptotic Bax and Bak proteins. Furthermore, the core proteins involved in apoptosis, including various death substrates such as PARP, were activated when treated with CXB and TAM in combination, which was consistent with the results of our apoptosis analysis.

VEGFR2 mediates Src regulation of endothelial cell junctions and vascular permeability [59,60]. Src proteins appear to be important for multiple aspects of tumor progression, including proliferation, disruption of cell-cell contacts, migration and invasiveness [61]. TAM and CXB additively reduced tumor migration and invasion; this finding was supported by our wound-healing and Boyden chamber assay results. We also demonstrated that CXB and TAM in combination interfered with the binding of VEGF to VEGFR2, thus suppressing the phosphorylation of Src protein and contributing to anti-metastatic activity leading to decreased MMP expression, as confirmed through the gelatin zymography and western blot analyses. Our study also showed that the ROS level decreased after co-administration of TAM and CXB confirmed through our FACS and in vivo studies.

Moreover, we proved the involvement of VEGFR2 signaling in the inhibition of Akt and MAPK molecules and in the phosphorylation of downstream proteins such as Bad and Bax, which play important roles in angiogenesis and apoptosis [24]. Supporting evidence concerning in vivo anti-angiogenic effects of TAM-CXB additively came from our chick embryonic CAM model and HUVEC-based tube formation assay with an in vitro model. All these results showed that treatment with both TAM and CXB suppressed the VEGFR2 pathways.

To thoroughly understand the extent of VEGF/VEGFR2 inhibition by TAM and CXB in combination, we performed VEGFR2 overexpression studies through luciferase assays and quantified the serum VEGF secretion levels. Results showed an approximately 3- and 2-fold increase in VEGFR2 promoter activity in transfected MCF7 and MDA-MB-231 cells, respectively. The observed VEGF-mediated up-regulation of VEGFR2 promoter activity in MCF7 and MDA-MB-231 cells was effectively suppressed by TAM and CXB in combination at very low concentrations (IC_50_ values) as compared with either drug alone. Finally, to validate the extent of VEGFR2 expression at mRNA levels, we performed RT-PCR studies and came up with similar results as the overexpression studies.

## Conclusion

In summary, our study indicated that the combination of TAM and CXB at nontoxic levels exerts potent anti-angiogenic effects by specifically targeting VEGF/VEGFR2 autocrine signaling through ROS generation. This additive combination suggests an effective approach with promising results in anti-metastatic and apoptotic studies. In a line, our preclinical studies suggest that this combination is a potential candidate treatment against breast tumors expressing high levels of VEGF and VEGFR2.

## Abbreviations

TAM: Tamoxifen; CXB: Celecoxib; VEGF-A: Vascular endothelial growth factor-A; VEGFR2: Vascular endothelial growth factor receptor 2; COX-2: Cycloxygenase-2; DCFDA: 2′,7′dichlorofluorescein diacetate; ROS: Reactive oxygen species; CAT: Catalase; SOD: Superoxide dismutase; RT PCR: Reverse transcriptase polymerase reaction; ER: Estrogen receptor; STAT3: Signal transducer and activator of transcription 3; MAPK: Mitogen-activated protein kinase; ELISA: Enzyme-linked immunosorbent assay; PARP: Poly(ADP-ribose) polymerase; HUVEC: Human umbilical vein endothelial cell; HIF-1α: Hypoxia-inducible factor 1α; PI: Propidium iodide; DAPI: 4′,6-diamidino-2-phenylindole; MTT: 3(4,5-dimethylthiazol-2-yl)-2,5 diphenyltetrazolium bromide; CBP: CREB-binding protein; VHL: von Hippel-Lindau tumor suppressor protein; MMP-2: Matrix metalloproteinase2; CAM: Chorioallantoic Membrane.

## Competing interests

No competing financial or personal interest in any company or organization is reported.

## Authors’ contributions

BNP, SR, MM conceived the study, designed the experiments, and drafted the manuscript. BNP and SR carried out the experiments. SD performed animal studies. KKD performed the RT-PCR studies. AP conducted VEGF quantification studies. AM and MM provided the critical revision of the manuscript. All authors read and approved the final manuscript.

## Pre-publication history

The pre-publication history for this paper can be accessed here:

http://www.biomedcentral.com/1471-2407/13/273/prepub
